# Evaluation of the effect of soybean diet on interferon-α-induced depression in male mice 

**Published:** 2017

**Authors:** Yazdan Azimi Fashi, Azadeh Mesripour, Valiollah Hajhashemi

**Affiliations:** 1 *Department of Pharmacology and Toxicology, School of Pharmacy and Pharmaceutical sciences, Isfahan university of Medical Sciences, Isfahan, Iran*; 2 *Isfahan Pharmaceutical Sciences Research Center, School of Pharmacy and Pharmaceutical sciences, Isfahan university of Medical Sciences, Isfahan, Iran*

**Keywords:** Interferon-α, Depression, Tryptophan, Soybean, Serotonin

## Abstract

**Objective:**

Interferon-α (IFN) therapy can cause depressive symptom which may lead to drug discontinuation. By interfering with tryptophan pathway, the available level of tryptophan required for serotonin synthesis decreases which could be related to depression. The aim of this study was to evaluate whether soybean diet could improve IFN-induced depression.

**Materials and Methods:**

Male mice weighing 28±3 g were used in the forced swimming test (FST) as an animal model of depression; also, locomotor activity was recorded. IFN 16×10^5 ^IU/kg was injected subcutaneously for 6 days. Animals were fed with regular diet or soybean diet at 3 concentrations throughout the experiment. Fluoxetine was the reference drug. To check whether the tryptophan content in the soy bean diet was effective, a group of animals was injected with a single dose of tryptophan on the test day.

**Results:**

IFN-α increased the immobility time in the FST (192 sec ± 5.4), that denotes depression in mice. Soybean diets caused less immobility that was more profound with 50% soybean (26.4 sec ± 6). This diet overcame the depression caused by IFN in the FST (54 sec±18). This result was parallel with that of tryptophan injected to animals (38 sec±17). All the animals showed normal locomotor activity.

**Conclusion:**

For the first time, we showed that soybean diet could counteract with depression caused by IFN-α. Since tryptophan therapy had similar effects, possibly the tryptophan content of soybean had induced the serotonin synthesis. Thus, not only less harmful kynurenine was produced but also more serotonin was available in the brain to overcome depression. However, this interpretation needs further evaluations.

## Introduction

In patients receiving cytokine treatments for viral infectious diseases (e.g. hepatitis) or several other types of malignancies, major depression develops frequently. Therapy with interferon α (IFN-α) causes severe adverse reactions; early signs include anorexia, fever and fatigue (Capuron et al., 2002a ▶), and prolonged therapy causes depressive symptoms in up to 45% of the patients that might lead to cessation of the therapy (Bonaccorso et al., 2002 ▶; Kraus et al., 2005 ▶). In animal studies, similar effects such as early signs of sickness (similar to human neurovegetative symptoms) were observed following IFN-α treatment (Sammut et al., 2001 ▶) and after chronic IFN-α treatment, depression-like behavior has been recognized (Makino et al., 2000 ▶; Ping et al., 2012 ▶). Simultaneous administration of imipramine reversed IFN-induced depression-like behavior in rats as assessed by forced swim test (Fischer et al., 2015 ▶).

Studies have proven some biological pathways that are involved in IFN-α-induced depressive symptoms. IFN-α can augment indoleamine 2,3-dioxygenase (IDO) enzyme activity; IDO is responsible for converting tryptophan to kynurenine. Therefore, kynurenine increases and the available concentration of tryptophan required for serotonin synthesis, decreases (Baranyi et al., 2013 ▶; Baranyi et al., 2015 ▶). Kynurenine can cross the blood-brain barrier and it breaks to metabolites such as quinolinic acid that is an agonist of N-methyl-D-aspartate (NMDA) receptor, and has neurotoxic effects (Wichers et al., 2005 ▶). 

Tryptophan is the precursor of serotonin (5-hydroxytryptamine, 5-HT), and tryptophan hydroxylase is responsible for hydroxylation of tryptophan to 5-hydroxytryptophan, which is the first and rate-limiting step in the synthesis of 5-HT. Serotonin has a prominent role in the neurobiology of mood disorders (Owens and Nemeroff, 1994 ▶). Reduced concentrations of tryptophan in blood are associated with reduced 5-HT accessibility within the central nervous system (CNS). Furthermore, low tryptophan levels has been related to the depressive relapse in vulnerable patients (Moore et al., 2000 ▶; Moreno et al., 2000 ▶). Previously, it has been shown that depressed patients who received no therapy, had lower plasma concentrations of tryptophan compared with control individuals (Ressler and Nemeroff, 2000 ▶; Song et al., 1998 ▶).

As noted earlier, IFN-α can affect mood probably by affecting biological pathways of tryptophan in the body. Soybean is a protein source comprised of tryptophan along with other amino acids (Banaszkiewicz, 2011 ▶), and for centuries, it has played a key role in the diet of many Asian countries (Yu et al., 2015 ▶). The positive influence of high protein products and tryptophan content of nutrients on depression, has been shown (Tavakkoli et al., 2015 ▶; Firk and Markus, 2009 ▶). Thus, the aim of the present study was to evaluate the effect of concomitant use of soy bean diet with IFN-α, to evaluate if an increase in amino acids especially tryptophan, in the diet could be beneficial on IFN-α induced depression. Although antidepressants have proven to be helpful on IFN-α-induced depressive behavior, but to the best of our knowledge, no study has analyzed the effect of soybean in this regard. Therefore, we used a mice model of depression to evaluate the effect of co-administration of IFN-α and different concentrations of soybean diets and also pure tryptophan treatment on depression.

## Materials and Methods

**Animals**

Male albino mice (weighing 28±3 g) were housed in cages, six animal in each cage at 21± 2 ºC with a 12 hr-12 hr light-dark cycle (the lights were on from 6 am to 6 pm). Tap water and standard mice chow were available, *ad libitum*. Tests were performed only after the mice had acclimated to the environment for at least 2 days. All experiments were performed between 08:00 and 13:00 hr in the pharmacology laboratory, in order to minimize circadian rhythm influence. For each treatment group, a minimum of six mice were used. All animal procedures were performed in accordance with guidelines for the Care and Use of Laboratory Animals Issued by Isfahan University of Medical Sciences, Isfahan, Iran (Grant No 395450).

**Locomotion test**

The motor activity of mice was assessed in a rectangular, plastiglas apparatus (Borj Sanat, Iran) divided by red beams into 15 zones in a 5×3 grid formation. Each zone was 9 ×10 cm. Mice were placed into the apparatus facing towards the wall in the closest corner to the experimenter and were allowed to explore the field for 3 min (Hemsley and Hopwood, 2005). The number of zone entries was counted automatically by passing animals through the beams and rears on hind-legs were recorded manually. The total activity was calculated by summing the zone entries (horizontal exploration) and rears (vertical exploration). 

**Forced swimming test (FST)**

Mice were forced to swim for 6 min in water of 25ºC in a glass beaker (diameter 12.5 cm). The depth was about 12 cm thus, the mice could not touch the bottom of the glass beaker with their paws or tail, and they could not escape. After 6 min, the mice were dried carefully and returned to their home cage. Behaviors that were recorded included: Immobility time, defined as the time spent while the animal was floating, staying still or using righting movements; recorded in the last 4 min. Swimming was defined as horizontal movements which involved at least two limbs (Bale and Vale, 2003 ▶). Latency to the first immobility was also recorded starting right after placing the mice in the water (Castagné et al., 2009 ▶). For each animal on the test day, first the locomotor activity and then, the FST was performed. 

**Drugs and Diets**

Interferon-α (IFN, Pooyesh Darou, 3×10^6^ IU), 16×10 ^5^ IU/kg body weight was injected subcutaneously for 6 consecutive days (dose was achieved according to pilot studies). Fluoxetine (Sigma-aldrich, Germany), as a selective serotonin inhibitor antidepressant, was given at the dose of 20 mg/kg (ip) on the last day, 30 min before the test (Kulkarni, 2007 ▶). Tryptophan (Sigma-aldrich, Germany), was given at the dose of 300 mg/kg (ip) one hour before the test (Cervo et al., 2005 ▶).

Soy beans (Brazilian) were purchased from Ziyar Soy Milk Company (Isfahan, Iran). Each 186 g row soy bean contained 68 g protein and about 1000 mg tryptophan. After soy beans were autoclaved for 30 min at 121ºC (Parsons et al., 1992 ▶), they were ground by a kitchen grinder. The powder was then carefully added to ground normal mice chow with the percentages of 20, 30, and 50 .

**Data processing and statistical analysis**

Results were expressed as group mean± SEM. All results were analyzed by one-way analysis of variance (ANOVA), followed by Tukey’s multiple comparison tests. p values less than 0.05 were considered significant. Excel 2010 and the Graphpad Prism 6 were used for data analyzing and making graphs.

## Results

**Figure 1:**
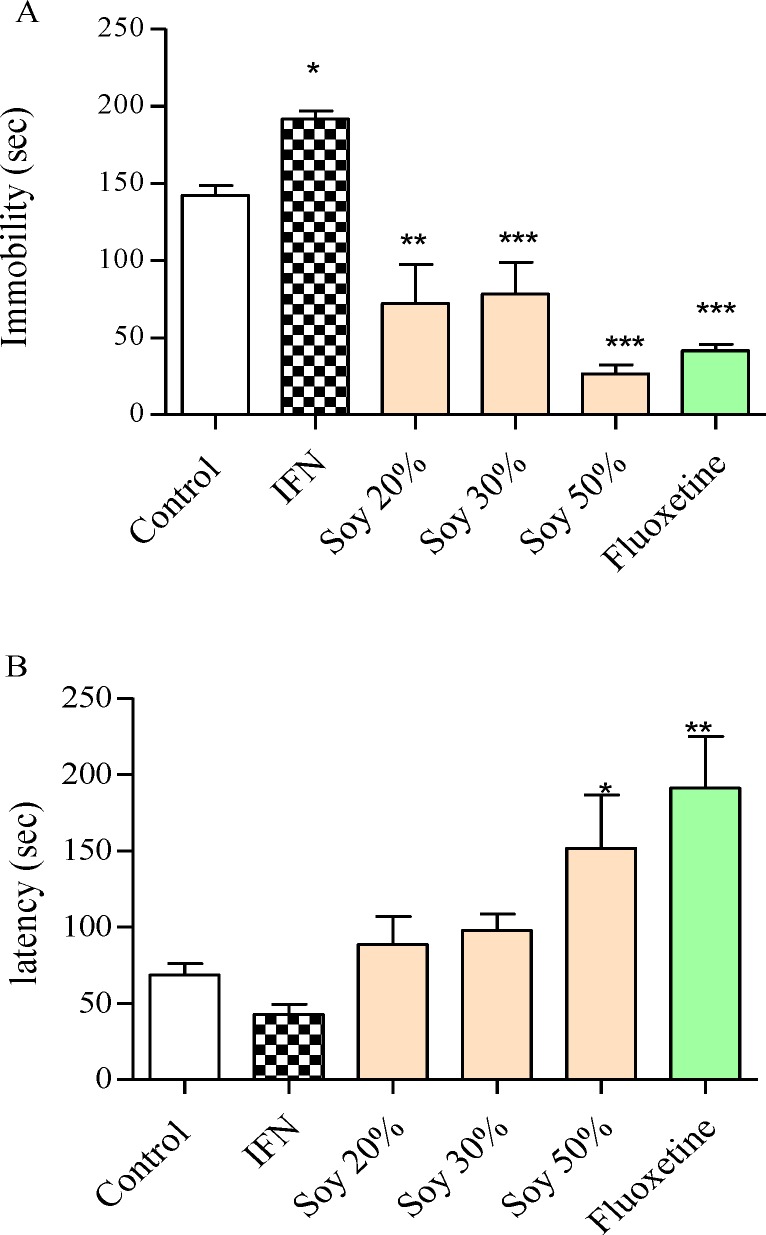
The effect of interferon-α (IFN) and different soybean diet concentrations on depressive performance in the forced swimming test. A) The effect of each diet on immobility time. B) The effect of each diet on the latency. The immobility time is the total time animals were immobile during the last 4 min of the total 6-min test. The latency time is the time spent until the animal becomes immobile. Number of animals in each group was 6. Control animals received normal mice chow. Results are expressed as group mean ± SEM and analyzed by ANOVA followed by Tukey’s comparison tests. * p<0.05 , ** p<0.01, and *** p<0.001 compared to the control group

Interferon-α considerably increased the immobility time in the FST (192 sec ± 5.4, p<0.05, f=25) compared to control (142 sec ± 6.5), which indicates the despair behavior induced by IFN-α (Figure 1A). As presented in Table 1, food consumption was not influenced by soybean diet and animals consumed soybean diet as the plane food chow diet. Administrating the soya diet to animals caused a decrease in the immobility time in the FST (Figure 1A). Immobility time significantly decreased by 50% soybean diet (26.4s ± 6, p<0.001, f=25) compared to the control group that consumed plane food chow (142 sec ± 6.5). The effect of soybean diet on immobility time was consistent with the changes that fluoxetine caused on the immobility time. Only 50% soybean diet significantly increased the latency to immobility (152 sec ± 35 vs 69 sec ± 7.6 control group, p<0.01, f= 5.9; Figure 1B). This change was similar to fluoxetine.

**Figure 2.**
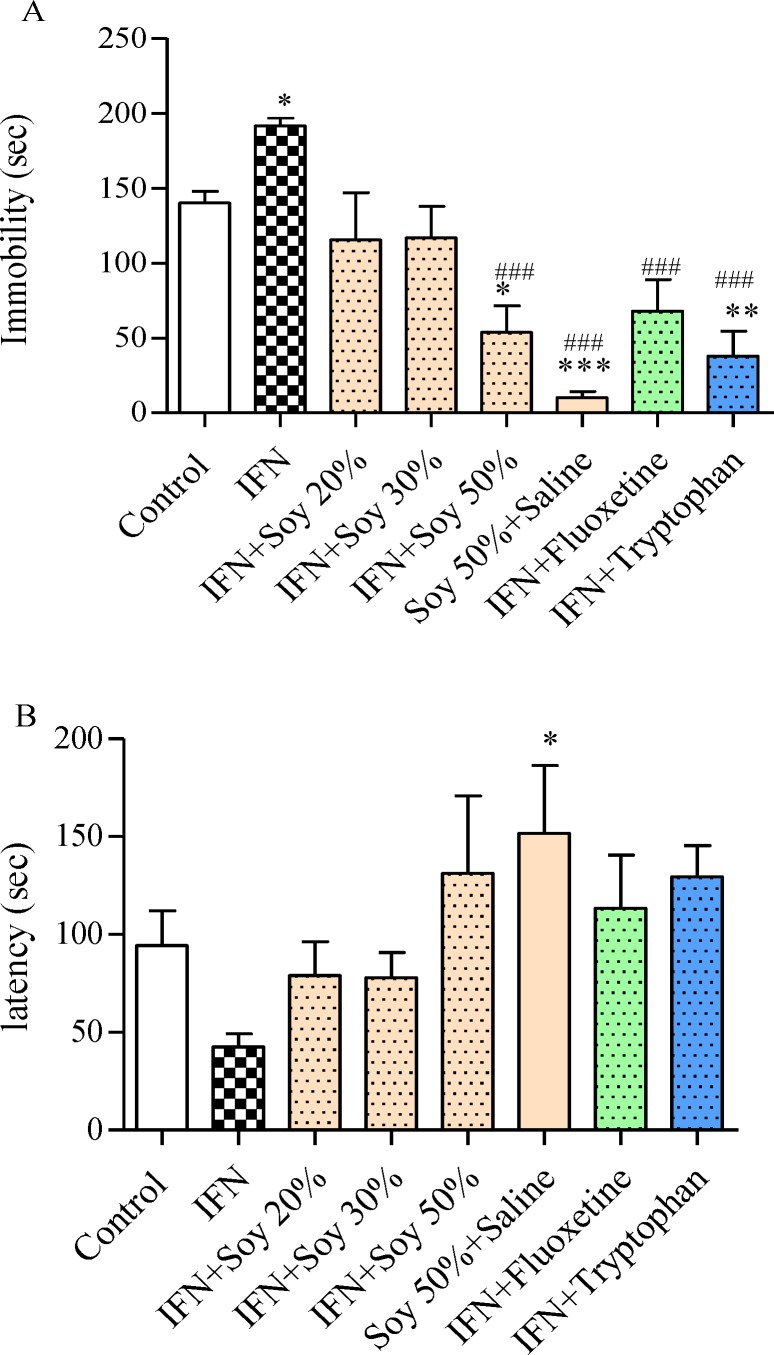
The effect of different soybean diet concentrations on interferon-α (IFN)-induced depressive performance in the forced swimming test. A) The effect of each diet on immobility time. B) The effect of each diet on the latency. The immobility time is the total time animals were immobile during the last 4 min of the total 6-min test. The latency time is the time it takes until the animal becomes immobile. IFN was administered (16×10^5 ^IU/kg, sc) after being diluted in saline, for 6 days, fluoxetine (20 mg/kg,ip) was administered 30 min before the test, tryptophan (100 mg/kg, ip) was administered one hour before the test. Number of animals in each group was 6. Control animals received normal mice chow. Results are expressed as group mean ± SEM and analyzed by ANOVA followed by Tukey’s comparison tests. * p<0.05, ** p<0.01, and *** p<0.001 compared to control group. ### p<0.001 compared to IFN

As Figure 2A shows, the immobility time significantly decreased (54 sec ± 18, p<0.05, f= 11) in the group that ingested 50% soybean diet concomitant with IFN, administrating tryptophan also mitigated the immobility time (38 sec ± 17, p<0.01, f= 11) versus IFN alone. Latency to immobility was increased by 50% soybean diet, tryptophan and fluoxetine concomitant treatment with IFN, although the difference was not significant (Figure 2B).

Table 2 presents the animals’ locomotor activity (vertical, horizontal, and total activities), no difference was seen amongst the groups and all treatment groups resulted in normal animal movements (number of total movements = 126.1 ± 7.1); thus, increased immobility of animals receiving IFN in the FST denotes the animal despair behavior.

**Table 1.** Food consumption during the test protocol

**Group**	**1** ^st^ ** 48 ** **(mg/g BW)**	**2** ^nd^ ** 48 (mg/g BW)**	**3** ^rd^ ** 48 (mg/g BW)**
**Control **	262 ± 28	283.5 ± 7.5	257 ± 8
**Soy 20%**	247.5 ± 30.4	263.3 ± 23.7	235.5 ± 24.1
**Soy 30 %**	242.2 ± 19.6	261.4 ± 7.6	272.1 ± 7
**Soy 50 %**	253.3 ±14.3	250.2 ± 21.7	287.3 ± 25.4
**IFN + Soy 20%**	303.8 ± 12.4	244.6 ± 12.2	242 ± 17.6
**IFN + Soy 30%**	331.8 ± 13.9	263.3 ± 12	279.3 ± 21.7
**IFN + Soy 50%**	201.4 ± 10.47	258 ± 13.7	218.1 ± 21.6

Control animals’ received plane mice chow, different diets were achieved by mixing 20, 30 or 50% soybean with normal food chow. Results are expressed as group mean ± SEM and analyzed by ANOVA followed by Tukey’s comparison tests

**Table 2:** The open field locomotor activity

**Group**	**Horizontal Movement**	**Vertical Movement**	**Total Movement**
**Control**	119.8 ± 5.6	6.2 ± 0.7	126.1 ± 7.1
**Soy 20%**	167.8 ± 9.9	12.8 ± 5.6	189.8 ± 8.4
**Soy 30%**	152.4 ± 12.9	16.5 ± 3	169 ± 13.3
**Soy 50 %**	167.8 ± 9.9	16.6 ± 1.7	184.5 ± 9.7
**IFN**	161.2 ± 5.1	17.1 ± 2.5	178.3 ± 5
**IFN + Soy 20%**	115.1 ± 9.1	9 ± 1.3	124.1 ± 9.4
**IFN + Soy 30%**	153.6 ± 10.4	12.6 ± 1.6	166.3 ± 10.5
**IFN + Soy 50%**	113.5 ± 17.3	11.7 ± 2.7	125.9 ± 22.6
**IFN+Tryptophan**	125.5 ± 6.9	12.3 ± 1.6	137.8 ± 7.5
**IFN+Flouxetine**	109 ± 5.6	10 ± 1	119 ± 5.4

The zone entries and hind-leg rears were counted for 3 min, the total activity is the sum of zone entries and rears. Number of animals in each group was 6. Control animals received normal mice chow. IFN was administered (16×10^5 ^IU/kg, sc) after being diluted in saline, for 6 days. Results are expressed as group mean ± SEM and analyzed by ANOVA followed by Tukey’s comparison tests

## Discussion

 In our study, IFN injection caused an increase in immobility time in the FST which was in favor of previous studies, indicating IFN-induced depression (Fischer et al., 2015 ▶). As the locomotor test revealed, total activity was not influenced by IFN; therefore, changes seen in the FST are mainly because of the animal despair behavior. Evidently depression occurs in patients who receive cytokine therapy for infectious diseases or cancers. Several mechanisms are involved in the effects of cytokine on behavioral such as the activation of inflammatory signaling pathways in the brain which causes the changes in monoamine, glutamate, and neuropeptide systems (Felger and Lotrich, 2013 ▶). It has been shown that the activity of inflammatory cytokines influences neurogenesis and neuroplasticity (Peng et al., 2008 ▶). Additionally, administrating IFN decreases systemic brain-derived neurotrophic factor (BDNF) levels in humans (Lotrich et al., 2013 ▶) and the reversal of apoptosis by antidepressants requires complete BDNF signaling (Peng et al., 2008 ▶). Among different mechanisms involved in IFN-induced depression, the tryptophan pathway was particularly considered in our study.

Previous research indicate that the severity of depressive symptoms was directly related to the decrease in tryptophan concentrations during treatment (Moreno et al., 2000 ▶). In patients undergoing cytokine therapy, a reduction in tryptophan availability, the precursor of serotonin, maybe the underlying cause for the development of depressive symptoms (Capuron et al., 2002b ▶). Soy bean contains various amino acids and a considerable amount of tryptophan. By using different soy bean concentrations in mice diet, antidepressant behavior was seen that was more obvious with the high soybean concentration in the diet. These findings were similar to those of tryptophan injection, thus, it could be concluded that higher tryptophan level could counteract with IDO induction caused by IFN in the kynurenine pathway (Baranyi et al., 2015 ▶).

Tryptophan is one of the essential amino acids that should be received through external sources since human body is unable to produce it (Heine et.al, 1995 ▶). In its free form, i.e. not albumin bond, it is transported across the blood-brain-barrier by the competitive and non-specific L-type amino acid transporter. Once in the CNS, tryptophan acts as a precursor in various metabolic pathways, which results in the production of proteins, serotonin and kynurenines (Ruddick et al., 2006 ▶). 

In the kynurenine pathway, the first stable intermediate product is kynurenine and the ultimate product is nicotinamide adenosine dinucleotide (NAD). Furthuremore, along the pathway, some other neuroactive intermediates are produced (e.g. quinolinic acid (NMDA receptor agonist), kynurenic acid (NMDA antagonist), and picolinic acid (neuroprotectant)) (Chen and Guillemin, 2009 ▶). Quinolinic acid concentration is normally lower in the brain in comparison to the peripheral tissues since tryptophan is metabolized to 5-HT rather than to formylkynurenine (Heyes et al., 1997 ▶). But, following an immune response, in the body and CNS, IDO becomes activated thus quinolinic acid concentration increases significantly. This same mechanism occurs when IFN is administered and IDO is activated.

In the brain, during inflammatory conditions, the major sources of quinolinic acid production are microglia and dendritic cells (Chen and Guillemin, 2009 ▶). However, astrocytes do not have the kynurenine hydroxylase enzyme; therefore, they are incapable of production of quinolinic acid (Guillemin et al., 2013 ▶). Neurons and astrocytes are protected because they uptake quinolinic acid and convert it to NAD. Unfortunately in pathological conditions, this process is saturated and the cells are not able to convert large amounts of quinolinic acid to NAD. Consequently, toxic amounts of quinolinic acid accumulate within the cells (Guillemin et al., 2005 ▶).

As our results showed, higher tryptophan concentration available either by tryptophan itself or by the soybean diet, could probably produce more 5-HT as compared to kynurenines; thus, not only less neurotoxins would be present but also more 5-HT is produced in the CNS. This could be the mechanism of the antidepressant effects observed here by the soybean diet concomitantly given with IFN that was similar to the reference drug, fluoxetine. However, in our study, tryptophan, kynurenines and their concentration ratio was not measured and it certainly warrants more investigations.

 The kynurenine pathway has been studied in various diseases, including major depression disorder (Laugeray et al., 2010 ▶). Studies have assessed the concentration of tryptophan and kynurenine in different disorders and imbalances were observed frequently. When tryptophan/kynurenines ratio were within normal ranges, the disease symptoms often alleviated (Chen and Guillemin, 2009 ▶). Therefore, it could be also deducted that by using soybean diet, tryptophan/kynurenines ratio returns to its normal levels thus IFN-induced despair behavior was relieved.

In conclusion, soybean diet could be considered as a harmless remedy for patients undergoing IFN treatment, and it is proposed to be further investigated against cytokine-induced depression.

## References

[B1] Bale TL, Vale WW (2003). Increased depression-like behaviors in corticotropin-releasing factor receptor-2-deficient mice: sexually dichotomous responses. J Neurosci.

[B2] Banaszkiewicz T, El-Shemy HA (2011). Nutritional value of soybean meal. Soybean and Nutrition.

[B3] Baranyi A, Meinitzer A, Breitenecker RJ, Amouzadeh-Ghadikolai O, Stauber R, Rothenhäusler HB (2015). Quinolinic acid responses during interferon-α-induced depressive symptomatology in patients with chronic hepatitis c infection-a novel aspect for depression and inflammatory hypothesis. PloS one.

[B4] Baranyi A, Meinitzer A, Stepan A, Putz-Bankuti C, Breitenecker RJ, Stauber R, Kapfhammer HP, Rothenhäusler HB (2013). A biopsychosocial model of interferon-alpha-induced depression in patients with chronic hepatitis C infection. Psychother Psychosom.

[B5] Bonaccorso S, Marino V, Puzella A, Pasquini M, Biondi M, Artini M, Almerighi C, Verkerk R, Meltzer H, Maes M (2002). Increased depressive ratings in patients with hepatitis C receiving interferon-α–based immunotherapy are related to interferon-α–induced changes in the serotonergic system. J Clin Psychopharmacol.

[B6] Capuron L, Gumnick JF, Musselman DL, Lawson DH, Reemsnyder A, Nemeroff CB, Miller AH (2002). Neurobehavioral effects of interferon-α in cancer patients: phenomenology and paroxetine responsiveness of symptom dimensions. Neuropsychopharmacology.

[B7] Capuron L, Ravaud A, Neveu P, Miller A, Maes M, Dantzer R (2002). Association between decreased serum tryptophan concentrations and depressive symptoms in cancer patients undergoing cytokine therapy. Mol psychiatry.

[B8] Castagné V, Porsolt RD, Moser P (2009). Use of latency to immobility improves detection of antidepressant-like activity in the behavioral despair test in the mouse. Eur J Pharmacol.

[B9] Cervo L, Canetta A, Calcagno E, Burbassi S, Sacchetti G, Caccia S, Fracasso C, Albani D, Forloni G, Invernizzi RW (2005). Genotype-Dependent Activity of Tryptophan Hydroxylase-2 Determines the Response to Citalopram in a Mouse Model of Depression. Neurobiol Dis.

[B10] Chen Y, Guillemin GJ (2009). Kynurenine pathway metabolites in humans: disease and healthy states. Int J Tryptophan Res.

[B11] Felger JC, Lotrich FE (2013). Inflammatory cytokines in depression: neurobiological mechanisms and therapeutic implications. Neuroscience.

[B12] Firk C, Markus CR (2009). Mood and cortisol responses following tryptophan-rich hydrolyzed protein and acute stress in healthy subjects with high and low cognitive reactivity to depression. Clin Nutr.

[B13] Fischer CW, Eskelund A, Budac DP, Tillmann S, ‌‌‌Liebenberg N, Elfving B, Wegener G (2015). Interferon-alpha treatment induces depression-like behaviour accompanied by elevated hippocampal quinolinic acid levels in rats. Behav Brain Res.

[B14] Guillemin G, Brew B, Noonan C, Takikawa O, Cullen K (2005). Indoleamine 2, 3 dioxygenase and quinolinic acid immunoreactivity in Alzheimer's disease hippocampus. Neuropathol Appl Neurobiol.

[B15] Guillemin GJ, Smith DG, Kerr SJ, Smythe GA, Kapoor V, Armati PJ, Brew BJ (2013). Characterisation of kynurenine pathway metabolism in human astrocytes and implications in neuropathogenesis. Redox Rep.

[B16] Hemsley KM, Hopwood JJ (2005). Development of motor deficits in a murine model of mucopolysaccharidosis type IIIA (MPS-IIIA). Behav Brain Res.

[B17] Heyes MP, Eugene O, Saito K (1997). Different kynurenine pathway enzymes limit quinolinic acid formation by various human cell types. Biochem J.

[B18] Heine W, Radke M, Wutzke KD (1995). The significance of tryptophan in human nutrition. Amino Acids.

[B19] Kraus MR, Schäfer A, Al‐Taie O, Scheurlen M (2005). Prophylactic SSRI during interferon alpha re‐therapy in patients with chronic hepatitis C and a history of interferon‐induced depression. J Viral Hepat.

[B20] Kulkarni SK, Dhir A (2007). Effect of various classes of antidepressants in behavioral paradigms of despair. Prog Neuropsychopharmacol Biol Psychiatry.

[B21] Laugeray A, Launay J-M, Callebert J, Surget A, Belzung C, Barone PR (2010). Peripheral and cerebral metabolic abnormalities of the tryptophan–kynurenine pathway in a murine model of major depression. Behav Brain Res.

[B22] Lotrich FE, Albusaysi S, Ferrell RE (2013). Brain-derived neurotrophic factor serum levels and genotype: association with depression during interferon-α treatment. Neuropsychopharmacology.

[B23] Makino M, Kitano Y, Komiyama C, Takasuna K (2000). Human interferon-α increases immobility in the forced swimming test in rats. Psychopharmacology.

[B24] Moore P, Landolt H-P, Seifritz E, Clark C, Bhatti T, Kelsoe J, Rapaport M, Gillin JC (2000). Clinical and physiological consequences of rapid tryptophan depletion. Neuropsychopharmacology.

[B25] Moreno FA, Heninger GR, McGahuey CA, Delgado PL (2000). Tryptophan depletion and risk of depression relapse: a prospective study of tryptophan depletion as a potential predictor of depressive episodes. Biol Psychiatry.

[B26] Owens MJ, Nemeroff CB (1994). Role of serotonin in the pathophysiology of depression: focus on the serotonin transporter. Clin Chem.

[B27] Parsons C, Hashimoto K, Wedekind K, Han Y, Baker D (1992). Effect of overprocessing on availability of amino acids and energy in soybean meal. Poult Sci.

[B28] Peng CH, Chiou SH, Chen SJ, Chou YC, Ku HH, Cheng CK, Yen CJ, Tsai TH, Chang YL, Kao CL (2008). Neuroprotection by Imipramine against lipopolysaccharide-induced apoptosis in hippocampus-derived neural stem cells mediated by activation of BDNF and the MAPK pathway. Eur Neuropsychopharmacol.

[B29] Ping F, Shang J, Zhou J, Zhang H, Zhang L (2012). 5-HT 1A receptor and apoptosis contribute to interferon-α-induced “depressive-like” behavior in mice. Neurosci lett.

[B30] Ressler KJ, Nemeroff CB (2000). Role of serotonergic and noradrenergic systems in the pathophysiology of depression and anxiety disorders. Depress Anxiety.

[B31] Ruddick JP, Evans AK, Nutt DJ, Lightman SL, Rook GA, Lowry CA (2006). Tryptophan metabolism in the central nervous system: medical implications. Expert Rev Mol Med.

[B32] Sammut S, Goodall G, Muscat R (2001). Acute interferon-α administration modulates sucrose consumption in the rat. Psychoneuroendocrinology.

[B33] Song C, Lin A, Bonaccorso S, Heide C, Verkerk R, Kenis G, Bosmans E, Scharpe S, Whelan A, Cosyns P, de Jongh R, Maes M (1998). The inflammatory response system and the availability of plasma tryptophan in patients with primary sleep disorders and major depression. J Affect Disord.

[B34] Tavakkoli-Kakhki M, Eslami S, Motavasselian M (2015). Nutrient-rich versus nutrient-poor foods for depressed patients based on Iranian Traditional Medicine resources. Avicenna J Phytomed.

[B35] Wichers M, Koek G, Robaeys G, Verkerk R, Scharpe S, Maes M (2005). IDO and interferon-α-induced depressive symptoms: a shift in hypothesis from tryptophan depletion to neurotoxicity. Mol Psychiatry.

[B36] Yu S, Guo X, Yang H, Zheng L, Sun Y (2015). Soybeans or soybean products consumption and depressive symptoms in older residents in rural Northeast China: A cross-sectional study. J Nutr Health Aging.

